# Cross-Border Meteorological Disaster Medical Rescue Policies in the Guangdong–Hong Kong–Macao Greater Bay Area: A Policy Text Quality Evaluation by PMC Index Model

**DOI:** 10.3390/healthcare14121617

**Published:** 2026-06-09

**Authors:** Hang Yang, Xi Wang, Tao Zhang, Rongjiang Cai, Shufang Zhao

**Affiliations:** 1 Faculty of Humanities and Social Sciences, Macao Polytechnic University, Macao, Chinaxwang@mpu.edu.mo (X.W.);; 2 School of Economics and Management, Ningbo University of Technology, Ningbo 315211, China

**Keywords:** cross-border medical rescue, meteorological disaster, policy evaluation, PMC index model

## Abstract

**Highlights:**

**What are the main findings?**

- The PMC index of the 26 policies averages 6.95, with 31% rated as excellent, but dimensions such as policy nature and timeliness score relatively low.
- Weak cross-border coordination mechanisms, shallow integration of medical professional content, and a weak link between short-term emergency response and long-term planning are the major deficiencies.

**What are the implications of the main findings?**

- Strengthening the functions of health departments and deepening medical professional integration in disaster rescue policies are urgently needed.
- Establishing a cross-regional joint evaluation and dynamic revision mechanism can improve policy coordination and long-term resilience in the Greater Bay Area.

**Abstract:**

**Background/Objectives:** Cross-border meteorological disaster medical rescue policies in the Guangdong–Hong Kong–Macao Greater Bay Area face challenges in coordination, completeness, and effectiveness. Existing policy systems lack systematic quantitative evaluation. This study aims to assess the current policy landscape and provide evidence-based recommendations for optimizing cross-border medical rescue policy supply and enhancing regional emergency coordination. **Methods:** We reviewed policy documents on cross-border meteorological disaster medical rescue issued from 2005 to 2025 and used a combination of text mining and the PMC index model to quantitatively analyze and evaluate selected policy texts. The PMC scoring criteria (0–10 scale) define scores ≥ 7 as “excellent” and 5–6.99 as “good”. **Results:** Policy word frequency analysis showed that “emergency,” “disaster,” “meteorology,” and “management,” were core high-frequency words; semantic network clustering revealed five major thematic modules: monitoring and early warning, emergency rescue, medical treatment, material support, cross-border coordination. The PMC indices of the 26 policies ranged from 5.65 to 9.42, with an average score of 6.95, which corresponds to the “good” level. Policy 14 scored 9.42, reaching the “perfect” level; eight policies received an “excellent” rating, indicating generally high policy quality. From a dimensional perspective, X9 (policy evaluation), X1 (Nature of policy), and X8 (policy guarantee) scored relatively high, while X4 (policy type) and X2 (policy timeliness) scored relatively low. **Conclusions:** The overall performance of the cross-border meteorological disaster medical rescue policy system is good, with relatively sound policy transparency and institutional guarantees. However, the policy system has the following shortcomings: insufficient cross-border coordination mechanisms, shallow integration of medical rescue professional content into comprehensive policies, and an emphasis on short-term emergency response with inadequate medium- and long-term strategic planning. It is recommended to strengthen medium- and long-term top-level strategic planning, enhance the functional allocation of health departments in meteorological disaster emergency plans, and establish a cross-regional joint policy evaluation and dynamic revision mechanism.

## 1. Introduction

Against the backdrop of intensifying global climate change, the cross-border spread of meteorological disasters has become a significant challenge threatening regional public health security and the lives and health of the people. Disasters such as typhoons, rainstorms, and extreme heat, due to their suddenness and destructiveness, pose a severe test to regional medical rescue systems [1,2]. A 2023 report showed that between 1990 and 2020, the risk of hospital damage caused by severe weather increased by 41% globally [3]. Valente et al. [4] found through a systematic review that floods and storms can severely disrupt hospital and pre-hospital services due to infrastructure damage and road closures, while heat waves significantly increase ambulance dispatch, emergency room visits, and hospitalizations, causing multi-dimensional impacts on the healthcare system. Climate change-driven extreme weather not only directly damages medical facilities and disrupts power and transportation, but also triggers a surge in emergency room demand, an imbalance between supply and demand of pre-hospital emergency resources, and a decline in the accessibility of long-term medical services [5,6]. Located on the southeastern coast of China, the Guangdong–Hong Kong–Macao Greater Bay Area is affected by both the monsoon climate and the marine environment, resulting in frequent meteorological disasters such as typhoons, rainstorms, and storm surges, which are characterized by high frequency, extreme nature, and complexity [7]. In 2024, meteorological disasters in the Greater Bay Area caused direct economic losses of approximately 2.092 billion yuan and 12 deaths, with the overall climate being relatively poor [8]. At the same time, due to the interconnectedness of the Greater Bay Area and the close exchanges between people, the cross-border transmission of meteorological disasters is particularly prominent. Once extreme meteorological disasters occur, they can easily cause cross-regional casualties and health threats, putting enormous pressure on cross-border medical rescue efforts.

As the forefront of opening up and the core area of the “one country, two systems” practice in China, the Guangdong–Hong Kong–Macao Greater Bay Area, relying on the unique pattern of “one country, two systems, and three currencies,” has significant differences in medical resource allocation, medical management systems, and medical insurance policies [9], which further increases the complexity of cross-border meteorological disaster medical rescue. In recent years, with the continuous advancement of the Greater Bay Area’s integration process, cross-border personnel flow and economic and trade exchanges have become increasingly frequent. The report shows that in 2023, the total population of the Guangdong–Hong Kong–Macao Greater Bay Area grew steadily, with the permanent resident population increasing by more than 440,000 [10]. Cross-border commuting, tourism, and employment activities have become more normalized, which has increased the region’s vulnerability under public emergencies and put forward higher requirements for rescue efficiency, coordination mechanisms, and policy guarantees [11,12].

China attaches great importance to the construction of the public health emergency response system in the Guangdong–Hong Kong–Macao Greater Bay Area, and has successively issued policy documents such as the “Outline Development Plan for the Guangdong-Hong Kong-Macao Greater Bay Area” and the “Implementation Plan for Further Improving the Medical and Health Service System in Guangdong Province,” which clearly propose to improve the regional emergency medical rescue linkage mechanism, promote the joint construction of medical consortia and regional medical centers with Hong Kong and Macao, and explore pilot projects for cross-border referral cooperation [13,14]. However, the current cross-border meteorological disaster medical rescue policies in the Greater Bay Area still have shortcomings, mainly manifested in the barriers and differences in medical management systems among the three places leading to poor coordination of rescue processes, uneven allocation of rescue resources, insufficient policy pertinence and operability, and difficulty in ensuring the smooth cross-border transfer of medical rights [15].

As a core tool of government governance, the completeness, coordination, and structural quality of policies directly affect the actual effectiveness of cross-border emergency response. In recent years, the academic community has paid increasing attention to emergency management policies, and the methods for quantitative evaluation of policies have been continuously enriched. Among them, the combined application of text mining and the Policy Modeling Consistency (PMC) index model, originally pioneered by Ruiz Estrada [16,17], provides an effective path for the systematic evaluation of policy systems. Ruiz Estrada’s foundational framework conceptualizes policy modeling as an omni-dimensional multi-input–output system, thereby overcoming the isolation and subjective bias inherent in traditional qualitative methodologies. To date, the PMC index model has been widely implemented to evaluate the structural robustness of China’s public policies, proving its high relevance to Chinese governance paradigms. For instance, in the realm of sector-specific and health-related planning, researchers have successfully utilized this approach to evaluate China’s pork industry policies [18], the strategic development policies for Traditional Chinese Medicine (TCM) [19], public health policy [20,21], management policy [22], and regional health promotion frameworks [23]. These diverse applications consistently demonstrate that the PMC model is uniquely capable of uncovering hidden structural gaps, policy tool imbalances, and horizontal inconsistencies within complex, multi-tiered Chinese administrative decrees. Consequently, the combination of text mining and the PMC framework helps to overcome the subjective limitations of traditional qualitative evaluation, providing empirical data support for optimizing the cross-border meteorological disaster medical rescue policy system.

In summary, the Guangdong–Hong Kong–Macao Greater Bay Area experiences frequent cross-border meteorological disasters and has an urgent need for cross-border medical rescue, while the existing policy system still has problems that urgently need optimization. Based on this, this paper takes the relevant policy texts on cross-border meteorological disaster medical rescue in the Guangdong–Hong Kong–Macao Greater Bay Area as the research object, and uses a combination of text mining and PMC index model to construct a policy evaluation index system, conduct quantitative analysis of policy quality, identify policy shortcomings, and provide theoretical support and practical reference for optimizing the policy system of cross-border meteorological disaster medical rescue in the Greater Bay Area and improving regional collaborative rescue capabilities.

## 2. Materials and Methods

### 2.1. Policy Text Mining and Analysis

**Figure 1:**
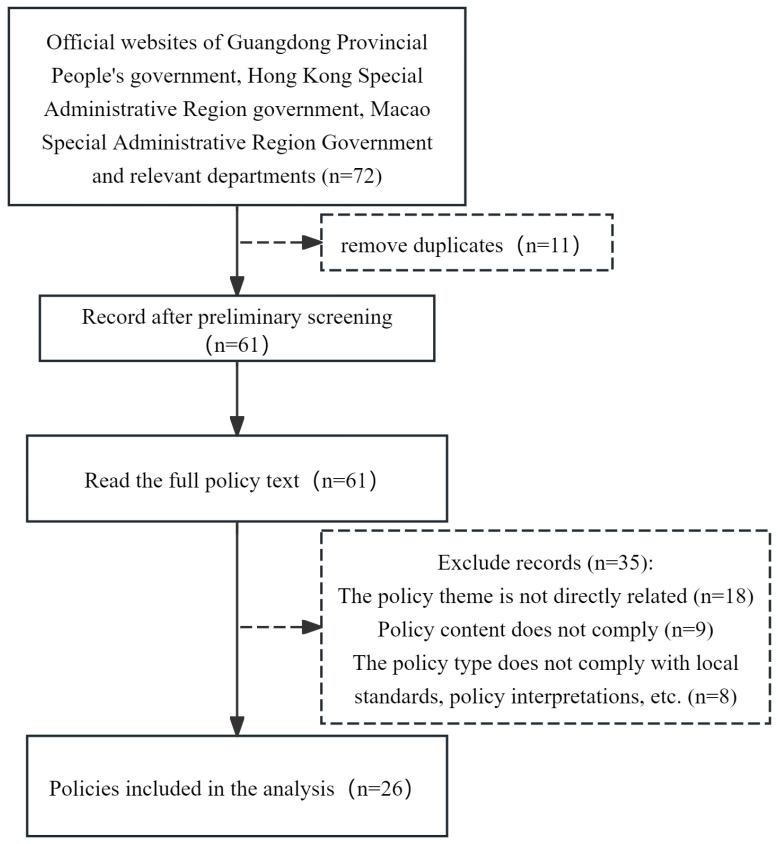
The policy text screening process.

To systematically retrieve policies related to cross-border meteorological disaster medical rescue, we conducted a comprehensive multi-stage policy search in March 2026, capturing documents issued between 2005 and 2025. Policy texts were harvested from the official web portals and relevant functional departments of the central government, Guangdong Province, the Hong Kong Special Administrative Region (HKSAR), and the Macao Special Administrative Region (MSAR) (e.g., the State Council, National Health Commission, and meteorological, emergency management, and security bureaus of the three regions). The advanced search engine of each database was queried using systematic Boolean combinations in both Chinese and English, formulated as follows: (“meteorological disaster” OR “typhoon” OR “rainstorm” OR “flood”) AND (“medical rescue” OR “health emergency” OR “medical treatment”) AND (“cross-border” OR “cross-boundary” OR “Guangdong-Hong Kong-Macao” OR “Greater Bay Area”) AND (“emergency plan” OR “contingency plan” OR “guideline” OR “protocol”). As illustrated in the policy screening flow (Figure 1), the initial strategic query yielded a pool of 72 raw records. After removing duplicates (*n* = 11), a total of 61 documents underwent rigorous preliminary full-text evaluation. Following the screening criteria, 35 documents were systematically excluded due to (1) policy theme not being directly related to cross-border scenarios (*n* = 18); (2) policy content not complying with primary evaluation objectives (*n* = 9); or (3) documents being secondary products such as policy interpretations, media briefing notes, or non-local administrative notices (*n* = 8). Ultimately, 26 highly relevant core policy documents were retained for quantitative analysis using the multi-input–output PMC index model. We used Rost CM 6 and Gephi 0.10.1 for word frequency analysis, semantic network analysis, and topic clustering. The 26 policy documents were converted into .txt format, retaining only the main bodies. Standard Chinese function words were removed using the Harbin Institute of Technology (HIT) Stop-word List, while generic administrative terms (e.g., “according to”, “department”, “notice”) were pruned using a custom stop-word dictionary. Synonyms were consolidated manually prior to software parsing: “cure” and “assistance” were merged into “rescue”; “healthcare” and “medical” into “medical”; and specific hazard terms (e.g., “flood/typhoon prevention”) into “disaster”. Words were ranked by absolute frequency, and the Top 100 high-frequency words were extracted to construct an initial symmetric co-occurrence matrix. To eliminate peripheral noise, a stringent edge filtering protocol was executed in Gephi, restricting the Edge Weight threshold to [108.0, 396.0]. Links below 108.0 were pruned, yielding a core network backbone of 57 nodes and 198 edges. Node size was mapped to its degree (scaled 1–4) to reflect centrality, and edge thickness was proportional to co-occurrence weight. Semantic clustering was executed using the Louvain modularity optimization algorithm (resolution = 1.0). Spatial rendering was visualized via the Fruchterman–Reingold layout algorithm with fixed control parameters: area = 10,000.0, gravity = 12.0, and speed = 1.0.

### 2.2. Construction of the PMC Index Model

This study uses the PMC index model to quantitatively evaluate the policy text of cross-border meteorological disaster medical rescue in the Guangdong–Hong Kong–Macao Greater Bay Area. By integrating high-frequency words and semantic clustering theme analysis in the policy text and combining the policy characteristics of cross-border meteorological disaster medical rescue in the Guangdong–Hong Kong–Macao Greater Bay Area, a policy evaluation index system is constructed. To ensure the objectivity of the scoring, two researchers independently evaluated 26 policies using PMC indicators and calculated the Cohen’s kappa coefficient to be 0.852 using Microsoft Excel. According to Landis and Koch’s (1977) criteria [24], this indicates a high degree of consistency among raters. The PMC model construction steps are mainly divided into four steps:

(1) Variable classification and parameter identification

First, determine the core evaluation dimensions and subdivide the specific indicators, and clarify the weight and scoring criteria of each parameter. Variables are constructed according to the cross-border meteorological disaster medical rescue policy text, including primary variables and secondary variables. All secondary variables have equal weight. The binary assignment method is used, as shown in Formulas (1) and (2), to assign the value of the secondary variables to 1 or 0:

X~N[0,1] (1)

X = {XR:[0~1]}(2)

(2) Establishing a multi-Input–Output table

Structure the parameter data to form a table showing the correspondence between inputs and outputs, ensuring the systematic nature of the evaluation. Using the above assignment results, combine the primary and secondary variables to construct a multi-input–output table for the policy text.

(3) Calculating the PMC index

First, calculate the value of each primary variable according to Formula (3). The value of a primary variable is determined by the arithmetic mean of its constituent secondary variables. Then, calculate the PMC index for each policy using Formula (4). In Formula (3), t represents the primary variable and j represents the secondary variable; in Formula (4), r represents the policy identifier and ~ represents the secondary variable. The multi-input–output table was structuralized, and all subsequent quantitative calculations—including the arithmetic means of primary variables and the summation of the final PMC indices for the 26 policies (Formulas (3) and (4))—were executed using Microsoft Excel.

(3) $$X_{t}=\frac{\sum_{j=1}^{n_{t}}X_{tj}}{n_{t}},\;(t=1,2,3,\ldots ,10)$$

(4) $$PMCr=\left[\begin{array}{c} X_{1}\left(\sum_{i=1}^{5}\frac{X_{1i}}{5}\right)+X_{2}\left(\sum_{j=1}^{3}\frac{X_{2j}}{3}\right)+X_{3}\left(\sum_{k=1}^{5}\frac{X_{3k}}{5}\right)+\\ X_{4}\left(\sum_{l=1}^{4}\frac{X_{4l}}{4}\right)+X_{5}\left(\sum_{m=1}^{10}\frac{X_{5m}}{10}\right)+X_{6}\left(\sum_{n=1}^{5}\frac{X_{6n}}{5}\right)+\\ X_{7}\left(\sum_{o=1}^{5}\frac{X_{7o}}{5}\right)+X_{8}\left(\sum_{p=1}^{6}\frac{X_{8p}}{6}\right)+X_{9}\left(\sum_{q=1}^{4}\frac{X_{9q}}{4}\right)+X_{10} \end{array}\right]$$

The PMC index ranges from 0 to 10, with higher values indicating better overall policy text quality and structural completeness. Referring to the classification criteria of Estrada et al., policies are divided into five levels: a PMC index ≥ 9.0 is considered a perfect policy; 7.0 ≤ PMC index < 8.99 is considered an excellent policy; 5.0 ≤ PMC index < 6.99 is considered a good policy; 3.0 ≤ PMC index < 4.99 is considered a fair policy; and a PMC index < 2.99 is considered an unsatisfactory policy (see Table 1).

(4) PMC Surface

To visually represent the differences in the performance of various policies across different dimensions, a PMC surface plot is used for visualization analysis. The PMC surface plot displays the score distribution of policies across each primary variable in a three-dimensional form. The degree of surface fluctuation reflects the balance and weaknesses of the policy structure. X10, as the policy disclosure dimension, is not included in the surface plot matrix due to its generally high score and limited discriminative power. The formula for constructing the PMC surface plot is shown in (5):

(5) $$PMCr=\left[\begin{array}{ccc} X_{1} & X_{2} & X_{3}\\ X_{4} & X_{5} & X_{6}\\ X_{7} & X_{8} & X_{9} \end{array}\right]$$

## 3. Results

### 3.1. Policy Text Mining Results

Through system screening, a total of 26 policy documents were collected. Table 2 details the policy title, year of publication, issue authority, and issue number. All 26 listed policies were retrieved from public and official governmental web portals. To facilitate reader access, the hyperlinked direct search repositories for these platforms are consolidated in Supplementary Table S1.

**Figure 2:**
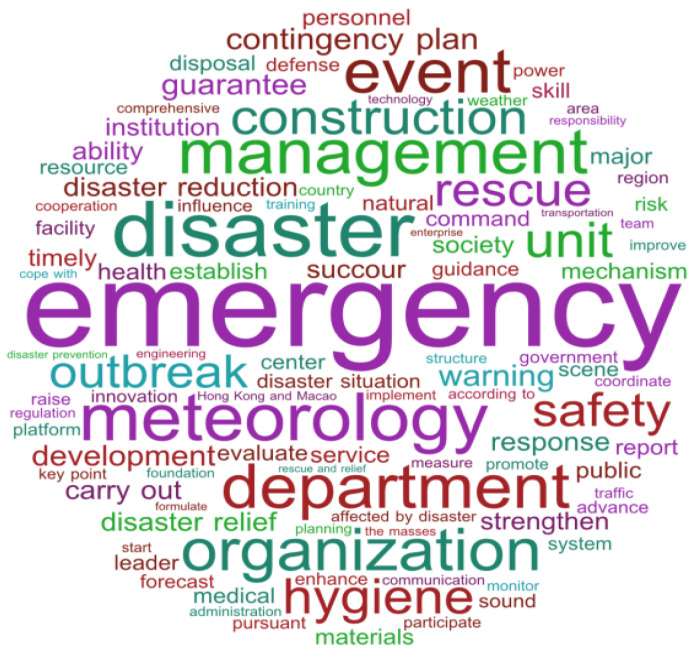
Frequency of words.

Word frequency analysis reflects the keywords frequently mentioned by policymakers. High-frequency terms extracted from policy texts help to gain a deeper understanding of the priorities and objectives proposed in cross-border meteorological disaster medical relief policies. In terms of data processing, 26 policy texts were imported into the system using the ROST CM for word frequency statistics. By removing irrelevant and generic words in this study, the top 100 high-frequency words were extracted. The first 50 words are presented in Table 3, and the complete list of 100 words is provided in Supplementary Table S2. The relevant high-frequency words were also visualized (in Figure 2).

**Figure 3:**
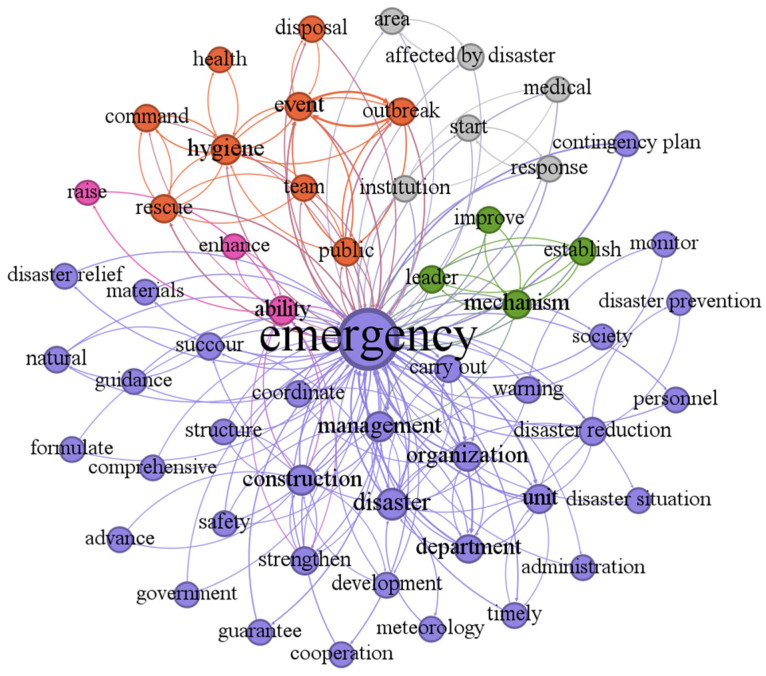
Thematic clustering map of semantic network.

High-frequency word co-occurrence network clustering diagrams are visualization tools used in text analysis to intuitively present lexical relationships and thematic structures. They use “nodes” to represent high-frequency words in the text, “lines” to represent co-occurrence relationships between words, and “clusters” formed by closely connected nodes to correspond to the core themes of the text. Based on the preprocessing and filtering protocols described in Section 2.1, a consolidated co-occurrence network with 57 nodes and 198 edges was generated Gephi, as shown in Figure 3.

To evaluate its structural characteristics and thematic cohesion, the network’s key topological indicators were computed: Graph Density = 0.062, Network Diameter = 4, Average Path Length = 2.156, Average Degree/Weighted Degree = 3.474/551.158, Average Clustering Coefficient = 0.500, and Modularity Index = 0.273. The modularity result indicates a statistically valid and stable community structure. By executing the Louvain algorithm under the Fruchterman–Reingold layout, the 57 core nodes were automatically partitioned into distinct classes. Based on the semantic properties of the clustered keywords, these modules were synthesized into five core policy themes: monitoring and early warning, emergency rescue, medical treatment, material support, and cross-border coordination.

### 3.2. Policy Indicator Evaluation System Results

This framework comprises 10 primary variables and 48 secondary variables. The primary variables include nature of policy (X1), policy timeliness (X2), policy field (X3), policy type (X4), policy focus (X5), policy receptor (X6), policy content (X7), policy guarantee (X8), policy evaluation (X9), and policy disclosure (X10). All secondary variables have equal weight and are assigned binary values. The complete evaluation indicator system is detailed in Table 4.

### 3.3. PMC Index Model Results

The results show that all 26 policies reached a good or above level, with an average PMC index score of 6.95. P14 performed exceptionally well, receiving a “Perfect” rating. Notably, 31% of the policies were rated “Excellent” due to their outstanding structural consistency. The policy ranking is as follows: P14 > P18 > P13 > P1 > P19 > P6 > P8 > P11 > P17 > P4 > P20 > P2 > P25 > P10 > P3 > P5 > P23 > P16 > P21 > P15 > P7 > P22 > P26 > P24 > P9 > P12.

Sensitivity analysis: Since all policies scored 1 on X10 (policy disclosure), this variable lacks discriminatory power and may artificially inflate the total PMC score. To assess its impact, we recalculated the PMC index excluding X10 (maximum possible score = 9) and linearly rescaled it to a 10-point scale. The results showed that the average score changed from 6.95 (including X10) to 6.61 (excluding X10), with an average difference of 0.34. The policy ranking remained virtually unchanged (Spearman’s ρ = 0.999), and the performance level (Perfect/Excellent/Good) of each policy stayed the same. Therefore, the inclusion of X10 does not bias the overall findings, and all substantive conclusions remain robust. To examine whether policy text quality has improved over time, we performed a Spearman rank correlation analysis between the enactment year and the PMC index score. Since multiple policies could be issued in the same year, we first averaged the PMC scores of policies within each year; the analysis yielded a correlation coefficient of (ρ = 0.645 *p* = 0.032), indicating a statistically significant positive association. This suggests that policies issued in more recent years tend to exhibit structural completeness and textual quality. Descriptive analysis further revealed that policies published after 2019—the year the “Outline Development Plan for the Guangdong-Hong Kong-Macao Greater Bay Area” was released—had a higher average PMC score (7.18) than those issued before 2019 (6.46). Although some recent local special plans (e.g., P2, P3) scored lower due to their narrow focus on single emergency responses, the overall upward trend remains evident. These findings indicate a gradual improvement in policy design quality from 2005 to 2025.

Due to layout limitations, Table 5 only displays the scores of various dimensions and PMC index for some policies (the complete data includes detailed score matrices for all 26 policies, see Supplementary Table S3). And Table 5 also shows a comparison of the PMC quantitative assessment scores for different types of policies: X1 (nature of policy), X3 (policy field), X5 (policy focus), X6 (policy receptor), X7 (policy content), X8 (policy guarantee), and X9 (policy evaluation) had higher average scores, while X4 (policy type) and X2 (policy timeliness) had lower average scores.

**Figure 4:**
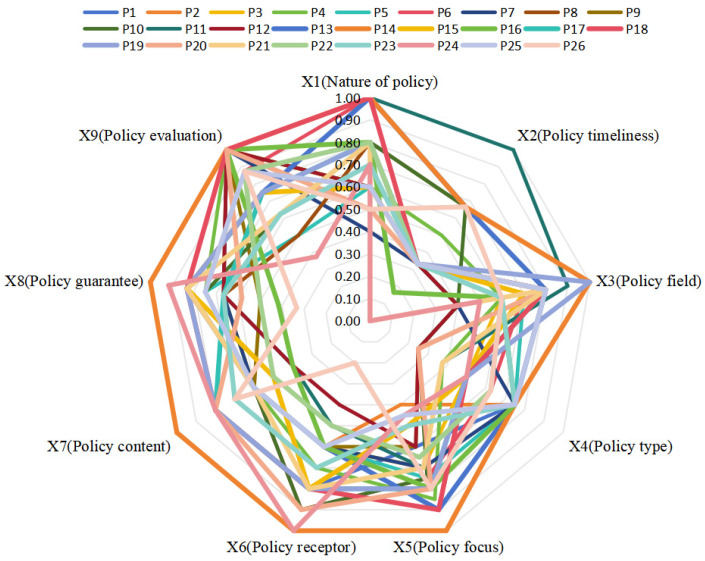
Radar chart of 26 policies.

The radar chart in Figure 4 visually presents the scores of the nine primary variables (X1–X9) for each of the 26 policies. Each axis in the radar chart represents one dimension, with values ranging from 0 to 1. Policies with a larger area and more balanced shape (e.g., P14) indicate strong and consistent performance across nearly all dimensions, whereas policies with irregular, indented shapes (e.g., P9, P12) reveal specific weaknesses, particularly in X2 and X4. By overlaying multiple policies, the radar chart allows for a quick cross-policy comparison of dimensional strengths and deficiencies.

**Figure 5:**
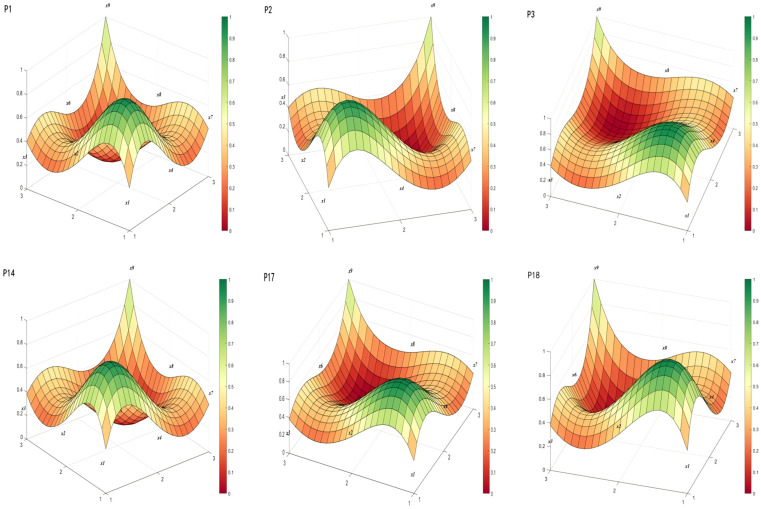
Policy excellence text quality surface.

To present the relevant policies more intuitively, we selected 6 out of the 26 policies (P1, P2, P3, P14, P17, P18) to display their PMC surface graphics, as shown in Figure 5, where the X and Y axes represent the coordinate matrix of indicators X1 to X9, and the Z axis denotes their respective scores. The smooth and elevated green terrain of top-level strategies like P14 demonstrates superior structural completeness and dimension coordination. Conversely, municipal-level or single-item protocols, such as P2 and P3, present sharply undulating landscapes dominated by expansive red and orange depressions, uncovering localized structural voids. This spatial configuration mathematically diagnoses that while the policy system achieves robust evaluation standardization (X9), it suffers from structural depressions in policy timeliness (X2) and tools supply (X4).

## 4. Discussion

### 4.1. Discussion of Text Mining Results

Word frequency analysis shows that emergency, disaster, meteorology, management, rescue, and cooperation are core words, indicating that the policy revolves around emergency response to meteorological disasters, medical rescue, and regional collaboration, which is highly consistent with the actual needs of cross-border joint prevention and control in the Guangdong–Hong Kong–Macao Greater Bay Area. The high frequency of Hong Kong and Macao reflects that cross-border cooperation has become a key issue in policymaking, reflecting the policy orientation of regional emergency integration under the “one country, two systems” principle. At the same time, the frequency of medical and health is stable, indicating that medical rescue has been embedded in the entire process of disaster emergency response, but compared with words such as emergency management and organization and command, its weight still has room for improvement. This reflects to some extent that the policy’s focus on the professionalization and process of cross-border medical rescue is still insufficient. Existing research has further confirmed this judgment [32], pointing out that although medical rescue has been included in the emergency management framework, in cross-border scenarios, the policy’s focus on the professional standards of medical rescue, cross-regional process connection, and institutional guarantee still needs to be strengthened.

The semantic network clustering diagram further reveals five major thematic modules of policy themes: First, “Medical Treatment,” forming the cluster with terms such as “health,” “hygiene,” “outbreak,” and “disposal” as nodes, emphasizing public health emergency responses; second, “Emergency Rescue,” encompassing keywords such as “response,” “start,” “medical,” and “institution,” reflecting operational rescue deployment and the activation of institutional protocols in affected areas; third, “Monitoring and Early Warning,” involving terms such as “mechanism,” “establish,” “improve,” and “leader,” which focus on institutional capacity building for upfront disaster tracking; fourth, “Material Support,” focusing on operational development with terms such as “ability,” “enhance,” and “raise”; and fifth, “Cross-border Coordination,” constituting the massive foundational network centered around the prominent node “emergency,” which incorporates terms like “management,” “coordinate,” “department,” “organization,” and “government,” reflecting the policy’s comprehensive institutional design for multi-departmental and cross-regional collaboration. These five clusters, to some extent, reflect the policy orientation of “prevention first, combined with prevention, resistance, and relief” in disaster governance system in China. Although there are connections between them, the co-occurrence strength between the two major clusters of “medical rescue” and “monitoring and early warning” is relatively weak, suggesting that current policy texts still have insufficient coordination in cross-domain integration such as cross-border medical transport, critical care, and public health prevention and control in meteorological disaster scenarios. A bibliometric study also shows that in recent years, research topics in the field of disaster medicine have mostly focused on the front-end and resilience building links such as “prevention-recovery-public health”, while the scale of medical rescue research is relatively small, which further confirms that there is still a structural gap in the current policy in terms of the integration of medical rescue and disaster prevention front-end [33].

From the perspective of disaster medicine and public health emergency response, meteorological disasters often lead to complex health risks such as outbreaks of infectious diseases and disruptions in chronic disease management [34,35]. Addressing these risks requires real-time conversion of meteorological monitoring data into medical rescue systems and coordinated responses. Existing research indicates that if meteorological warning information is not effectively embedded in the health emergency decision-making mechanism, it will lead to problems such as delayed allocation of rescue resources and low efficiency in cross-regional transfer of critically ill patients [36]. From a discourse perspective, policy texts frequently use verbs such as “strengthen,” “establish,” “improve,” and “implement,” reflecting the policymakers’ intention to enhance emergency response capabilities primarily through the formulation of systems. This provides a necessary institutional framework and policy basis for regional collaborative governance, while also reserving space for subsequent formulation of specific operational rules, clarification of responsible entities, and resource allocation processes. In actual emergency response, how to effectively transform such macro-level institutional designs into executable cross-departmental and cross-border collaborative solutions remains a crucial aspect that needs further focus in the policy improvement process.

The policies released between 2019 and 2025 coincide with the timeline of accelerated regional collaboration following the release of the “Outline Development Plan for the Guangdong-Hong Kong-Macao Greater Bay Area,” demonstrating timely policy response. However, the number of policies from 2005 to 2018 was small, and the early documents mainly focused on intra-provincial emergency response, lacking content on cross-border medical rescue, reflecting a historical gap and untimely updates in the policy system. Chen et al. pointed out [37] that the policy window period helps improve the efficiency of cross-departmental collaboration in flood disaster management in the Greater Bay Area, while insufficient institutional continuity will hinder the continuous accumulation of collaborative capabilities.

### 4.2. Discussion of PMC Index Model Results

The 26 policies showed good overall effectiveness, with an average PMC index of 6.95, all reaching a good or above level. This finding echoes the quantitative evaluation results of China’s disaster relief policies by Li et al. [38]—the evaluation of ten representative disaster relief policies showed that the PMC indices of the selected ten policies were all above acceptable levels, and the scores of highly authoritative policies were generally higher than those of specific policies. Among them, P14 received a perfect rating, and P18, P13, P1 and others were rated as excellent, indicating that the top-level planning, provincial special projects, and regional cooperation policies were more scientifically designed and had more complete elements. The scores of municipal-level plans (P2, P3) and single-item relief plans (P9, P12) were relatively low, reflecting that the policy design quality and text completeness decreased as it descended to the next level, and that the grassroots policies lacked adaptability and operability for cross-border meteorological disaster medical relief.

From the average scores of the 26 policies across various dimensions, policy evaluation (X9) scored the highest, indicating that the policies are well-founded, have clear objectives, and well-defined responsibilities, demonstrating strong standardization in their formulation. However, this robust textual standardization and clear definition of institutional mandates within X9 must not be loosely interpreted as a guarantee of practical implementation success. While the policy texts successfully outline structured goals and programmatic rationales on paper, the conversion of these formal criteria into effective real-world rescue actions remains highly contingent upon dynamic administrative coordination and local enforcing capabilities across borders. Nature of policy (X1) and policy guarantee (X8) also scored highly, indicating that the policies possess multiple functions such as prediction, supervision, and guidance, and that basic guarantee elements such as finance, facilities, talent, and healthcare are covered. However, policy timeliness (X2) scored the lowest, indicating that most policies focus on short-term emergency response, with insufficient coverage of medium- and long-term (3–5 years) and long-term (≥5 years) planning. There is a lack of continuous arrangements for cross-border medical rescue capacity building, facility layout, and technological research and development, making it difficult to support the long-term resilience of the region. Policy type (X4) also scored low. Further analysis revealed that existing policies are mainly “constructive” and “guiding,” emphasizing infrastructure construction, capacity building, and direction advocacy, while the application of “command-type” and “incentive-type” policy tools is significantly insufficient. Insufficient rigid constraints lead to weak cross-border cooperation implementation, and the lack of incentive mechanisms makes it difficult to mobilize the enthusiasm of medical institutions and social forces to participate. Xu et al. [39] quantitative evaluation of the emergency response plans for public health emergencies of Chinese provincial governments also found that the existing emergency plans have obvious deficiencies in terms of monitoring and early warning, early response, emergency support, recovery and reconstruction, working mechanisms and plan reliability.

Some policies scored poorly in terms of cross-border special focus and professionalism in medical rescue, indicating that the policy design for coupling the three scenarios of meteorological disasters, cross-border, and medical rescue is insufficient, and there are still deficiencies in the linkage mechanism, responsibility boundaries, and disposal procedures. Xu et al. [40] PMC evaluation of emergency supplies policies in the Yangtze River Delta also revealed similar problems: the design consistency of mechanism-oriented dimensions (such as linkage response) is significantly lower than that of policy objectives, and the overall design quality of cross-regional coordination mechanisms is uneven. This discrepancy highlights a fundamental methodological distinction within policy analysis: high textual consistency across dimensions does not automatically translate into active implementation synergy. Under the “one country, two systems” framework in the Greater Bay Area, even if top-level policies (such as P14 or P18) achieve excellent structural completeness scores in the PMC matrix, their real-world operationalized impacts are frequently restricted by institutional friction, differing legal jurisdictions, and fragmented resource authorities. Therefore, the “good” and “excellent” text quality levels identified in this study are interpreted as robust institutional blueprints and necessary prerequisites for collaboration, rather than being directly equated with successful on-the-ground crisis alleviation or optimized health outcomes.

### 4.3. Comprehensive Analysis of Text Mining and PMC Evaluation

A comprehensive analysis of text mining and PMC evaluation results reveals a core contradiction: while there is a high degree of consensus on cross-border collaboration at the discourse level, its institutional embedding is insufficient. In the semantic network clustering diagram, words such as cooperation, coordination, and Guangdong–Hong Kong–Macao Greater Bay Area form obvious cluster nodes, indicating that cross-border issues occupy a certain position in policy discourse. However, from the perspective of the X3 and X7 secondary variables in the PMC evaluation, the specific clauses involving cross-border collaboration generally have low values assigned to key institutional elements such as response initiation conditions, division of responsibilities among the three regions, and joint command mechanisms. Most policy texts focus on principled statements such as strengthening cooperation, establishing mechanisms, and information sharing, lacking operable and accountable institutional designs. It is crucial to emphasize that the frequent appearance of terms such as “cooperation”, “coordination”, and “mechanism” in policy texts reflects a discursive consensus at the formulation stage but does not constitute evidence of their operational effectiveness in real cross-border rescue scenarios. The gap between textual articulation and practical implementation remains substantial, and future empirical research is required to assess how these well-intentioned provisions actually function under the institutional complexities of the “one country, two systems” framework.

This gap between consensus and institutional framework reflects the deep-seated challenges faced by Guangdong, Hong Kong, and Macao in the field of cross-border medical rescue. Under the “one country, two systems” framework, the three regions have institutional differences in legal systems, administrative systems, and medical resource management authority, making it difficult to promote cross-border collaboration through a single administrative order or standardized policy tool [41]. Existing policy texts tend to reach consensus on soft cooperation while leaving gaps in hard institutional frameworks. While this approach helps avoid institutional conflicts, it also leaves cross-border relief operations lacking clear responsibilities and operational pathways. The high frequency of terms like “mechanism” and “coordination” in text mining, coupled with their low semantic network nodalism, further confirms that cross-border collaboration is positioned more as a background issue than a core module within the policy framework.

### 4.4. Policy Implications and Recommendations

Based on the comprehensive analysis of the above text mining and PMC index model, the following optimization suggestions are proposed to address the structural shortcomings of the cross-border meteorological disaster medical rescue policy system in the Guangdong–Hong Kong–Macao Greater Bay Area:

To overcome the limitation of current policies being overly focused on short-term emergency deployment, policymakers should strengthen medium- and long-term strategic planning and top-level designs. Future policy formulations should incorporate 3–5 year and longer-term development blueprints, specifying continuous arrangements for cross-border medical rescue capacity building, specialized facility layouts, and joint technological research and development, thereby enhancing the long-term resilience of the region. At the same time, we should enrich the types of policy tools, introduce command-based and incentive tools, clarify the responsibility boundaries and accountability mechanisms of the governments and departments in the three regions in cross-border rescue, set binding provisions, and provide substantive incentives for medical institutions and social organizations to participate in rescue, such as resource preference, financial subsidies, assessment and evaluation. In addition, we should promote the integration of meteorological and medical policies, establish the corresponding relationship between the meteorological disaster early warning level and the medical rescue response level, and form the presetting and activation mechanism of medical forces after the early warning is issued.

### 4.5. Limitations and Future Research Directions

This study has several limitations. First, the policy sample is limited to 26 publicly released policy documents from 2005 to 2025. While this covers representative policies at the national, provincial, and municipal levels, as well as cross-border cooperation, there may still be policy omissions, particularly the exclusion of some unpublished internal operational documents or inter-departmental agreements, which could affect the completeness of the policy system. Second, the PMC index model uses a binary assignment method with equal weights for each secondary variable. While this ensures objectivity and operability, it fails to reflect the differences in importance of different policy elements in actual implementation, which represents a well-recognized intrinsic constraint in standard PMC methodological applications [16,17]. Third, this study focuses strictly on the completeness, consistency, and structural design of the policy texts through the PMC index model, without tracking or measuring actual policy implementation effects, implementation processes, or on-site rescue effectiveness. Consequently, the “high scores” obtained by certain policies signify robust institutional design and textual completeness on paper, but a gap may still exist between high-quality textual formulation and real-world impact. As substantiated by empirical policy evaluation, high consensus during the text formulation stage does not automatically yield operational efficacy, necessitating downstream field studies to bridge this text–practice divergence [38]. Additionally, the correlation analysis between policy year and PMC index, while revealing a significant positive association (ρ = 0.645, *p* = 0.032), is based on a limited number of annual data points after averaging policies within the same year. The small sample size and uneven temporal distribution warrant cautious interpretation.

Future research could be deepened and expanded in the following directions: First, broaden the policy sample coverage by further collecting supporting implementation rules and operational guidelines formulated by health, emergency management, and meteorological departments in the three regions to construct a more complete policy dataset. Second, by combining empirical case studies, post-implementation field evaluations, or stakeholder interviews, empirical studies will be conducted on the actual implementation, obstacles, and execution effects of typical policies to bridge the gap between textual evaluation results and real-world rescue effectiveness. Third, multi-dimensional policy evaluation methods will be explored, and the weighting of PMC indicators will be optimized by introducing expert empowerment and the analytic hierarchy process to make the evaluation results more closely reflect policy practice. Fourth, future research with larger policy datasets and longer time spans is needed to further confirm the robustness of this temporal trend and to explore the causal mechanisms underlying the improvement in policy design quality.

## 5. Conclusions

This study employs text mining and the PMC index model to quantitatively evaluate 26 cross-border meteorological disaster medical rescue policies in the Guangdong–Hong Kong–Macao Greater Bay Area from 2005 to 2025. The results show that the current policy system is emergency-oriented, forming five major thematic modules (monitoring and early warning, emergency rescue, medical treatment, material support, cross-border coordination), but the semantic connection between meteorology and medicine remains weak, indicating insufficient cross-domain integration. It should be noted, however, that the high frequency of terms such as “coordination” and “mechanism” in policy texts does not automatically guarantee their effective implementation; bridging the text–practice gap remains a critical challenge. The PMC evaluation yields an average score of 6.95 for 26 policies, suggesting a generally good to excellent level, with high scores in policy evaluation, nature, and guarantee, but relatively low scores in policy timeliness and type. Overall, the cross-border meteorological disaster medical rescue policy system demonstrates good textual quality and structural completeness, with relatively sound policy transparency and institutional guarantees. This reflects structural shortcomings: a dominance of short-term emergency response with inadequate medium- and long-term planning and capacity building, and an over-reliance on constructive and guiding policy tools while command-based and incentive-based tools are underutilized. The study further reveals that cross-border collaboration enjoys high consensus at the discourse level but is insufficiently embedded institutionally. Key institutional elements—such as response activation, responsibility allocation, and joint command—remain largely principled statements lacking operational and accountable design, rooted deeply in the institutional differences under the “one country, two systems” framework. In summary, while the overall text quality and structural completeness for cross-border meteorological disaster medical rescue in the Greater Bay Area is relatively sound, improvements are needed in enhancing medium- and long-term strategic planning supply, optimizing policy tool structures, institutionalizing cross-border coordination, and integration between meteorological and medical policies. Future research could expand policy samples, incorporate implementation process evaluation, and conduct cross-border comparative studies to deepen empirical analysis of actual policy impact and collaborative mechanisms.

For policymakers in the Greater Bay Area, three actions are recommended: (1) strengthen medium- and long-term strategic planning to overcome the current over-reliance on short-term emergency responses; (2) introduce binding command-type and incentive-type policy tools, such as accountability clauses and financial rewards, to operationalize cross-border coordination; and (3) establish a graded response protocol that directly links meteorological warning levels with medical rescue dispatch triggers, thereby closing the current gap between weather monitoring and health action.

## Figures and Tables

**Table 1:** Policy scoring levels.

Evaluation Grade	Poor	Acceptable	Good	Excellent	Perfect
PMC score	0–2.99	3–4.99	5–6.99	7–8.99	9–10

**Table 2:** Summary of samples for evaluating the text quality of policies.

No.	Title	Issuing Authority	Issue Number	Year
P1	Regulations of Guangdong Province on Promoting Meteorological Cooperation and Development among Guangdong, Hong Kong and Macao	Standing Committee of the People‘s Congress of Guangdong Province	No. 62	2025
P2	Meizhou City Rainstorm Disaster Warning and Response Regulations	Standing Committee of the People’s Congress of Meizhou City	No. 26	2025
P3	Notice of the Guangzhou Municipal Health Commission on Issuing the Emergency Response Plan for Medical and Health Rescue for Flood Control, Drought Relief, Typhoon Prevention and Freezing Prevention	Guangzhou Municipal Health Commission	/	2025
P4	Zhuhai City Natural Disaster Relief Emergency Plan	Zhuhai Municipal People’s Government Office	Zhuhai Municipal Government Office Letter No. 33 [2025]	2025
P5	Guangzhou City Natural Disaster Relief Emergency Plan	Guangzhou Municipal Disaster Reduction Committee Office	/	2024
P6	Cooperation Agreement on Emergency Response Mechanisms for Public Health Emergencies Caused by Infectious Diseases among Mainland China, Hong Kong and Macao	Health Bureau of the Macao Special Administrative Region Government	/	2024
P7	Guangdong Province Natural Disaster Relief Emergency Plan	General Office of the People’s Government of Guangdong Province	Guangdong Provincial Government Office Letter No. 250 [2024]	2024
P8	Framework Agreement on Cooperation in Emergency Management and Emergency Rescue Operations in the Guangdong-Hong Kong-Macao Greater Bay Area	Office of the Secretary for Security of the Macao Special Administrative Region	/	2024
P9	Notice on Issuing the Emergency Response Plan for Medical and Health Rescue in Guangdong Province	General Office of the People’s Government of Guangdong Province	Guangdong Provincial Government Office Letter No. 18 [2024]	2024
P10	Shenzhen Natural Disaster Relief Emergency Plan (2024 Revision)	Shenzhen Emergency Management Bureau	Shenzhen Disaster Reduction Committee No. 1 [2024]	2024
P11	Shenzhen Emergency Response Plan Management Measures	Shenzhen Emergency Management Bureau	Shenzhen Emergency Management Bureau Document No. 4 [2023]	2023
P12	Notice on Issuing the Emergency Response Plan for Meteorological Disasters in Guangdong Province	Guangdong Provincial People’s Government	Guangdong Provincial Government Letter No. 329 [2021]	2021
P13	Notice on Issuing the Implementation Opinions of Guangdong Province on Promoting the Construction of a Pilot Demonstration Province for the First Line of Defense in Meteorological Disaster Prevention and Mitigation	General Office of Guangdong Provincial Government	Guangdong Provincial Government Office Letter No. 311 [2021]	2021
P14	Notice on Issuing the 14th Five-Year Plan for Emergency Management in Guangdong Province	Guangdong Provincial People’s Government	Guangdong Provincial Government Document No. 67 [2021]	2021
P15	Guangdong Province Regulations on Lightning Disaster Prevention	Guangdong Provincial People’s Government	The People’s Government of Guangdong Province No. 284	2021
P16	Guangdong Province Emergency Plan for Flood Control, Drought Relief, Typhoon Prevention and Freezing Prevention	General Office of Guangdong Provincial Government	Guangdong Provincial Government Office Letter No. 54 [2021]	2021
P17	Meteorological Development Plan for the Guangdong-Hong Kong-Macao Greater Bay Area (2020–2035)	China Meteorological Administration	/	2020
P18	Ten-Year Plan for Disaster Prevention and Mitigation of the Macao Special Administrative Region (2019–2028)	Macao Special Administrative Region Government	/	2019
P19	Outline Development Plan for the Guangdong-Hong Kong-Macao Greater Bay Area	CPC Central Committee and State Council	No. 7 [2019]	2019
P20	Consensus on Health Cooperation in the Guangdong-Hong Kong-Macao Greater Bay Area	Health Commission of Guangdong Province, Food and Health Bureau of Hong Kong Special Administrative Region Government, Health Bureau of Macao Special Administrative Region Government	/	2019
P21	Guangdong Provincial Regulations on Meteorological Disaster Prevention	Standing Committee of the People‘s Congress of Guangdong Province	No. 27	2014
P22	Notice on Issuing the Measures for the Management of Emergency Response Plans of Guangdong Province	General Office of the People’s Government of Guangdong Province	Guangdong Provincial Government Office Document No. 36 [2008]	2008
P23	Notice Forwarding the Notice of the General Office of the Ministry of Health on Strengthening Health Emergency Response to Floods and Other Natural Disasters	Guangdong Provincial Health Commission	Guangdong Provincial Health Commission Office Document No. 54 [2008]	2008
P24	Shenzhen Emergency Response Plan for Public Health Emergencies	Shenzhen Municipal People’s Government General Office	Shenzhen Municipal Government Office Document No. 148 [2007]	2007
P25	Emergency Notice of the Provincial Patriotic Health Campaign Committee on Conscientiously Carrying Out Patriotic Health Work During Floods and Post-Disaster Recovery	Guangdong Provincial Patriotic Health Campaign Committee	Guangdong Patriotic Health Campaign Document No. 11 [2006]	2006
P26	Zhuhai City General Emergency Response Plan for Public Emergencies	Zhuhai Municipal People’s Government	Zhuhai Municipal People’s Government Order No. 50	2005

**Table 3:** Top 50 high-frequency word list.

Words	Frequency	Words	Frequency	Words	Frequency	Words	Frequency
emergency	3040	response	551	outbreak	637	materials	390
disaster	1271	succour	523	contingency plan	631	medical	371
meteorology	946	ability	506	warning	626	command	371
management	843	strengthen	488	guarantee	595	disposal	362
department	811	institution	483	development	588	natural	362
event	802	society	455	disaster reduction	566	disaster situation	360
organization	748	timely	454	carry out	564	facility	358
construction	741	service	416	disaster relief	554	guidance	357
unit	737	public	414	emergency	3040	response	551
hygiene	729	establish	406	disaster	1271	succour	523
rescue	674	personnel	401	meteorology	946	ability	506
safety	661	mechanism	400	management	843	strengthen	488

**Table 4:** Scoring criteria for secondary variables.

Primary Indicators	Secondary Indicators	Variables	Scoring Criteria	Reference Source
Nature of policy	X1	(X1:1) Prediction	1. Does the policy assess trends in disaster prevention and mitigation?	[25]
		(X1:2) Supervision	2. Does the policy include oversight of cross-border relief operations, resource allocation, and departmental responsibilities?	[26]
		(X1:3) Suggestions	3. Does the policy provide guidance, suggestions, or non-mandatory measures?	
		(X1:4) Feedback	4. Does the policy clearly define feedback mechanisms for the effectiveness of various policy initiatives?	
		(X1:5) Guidance	5. Does the policy include guidance on disaster prevention and mitigation, such as technical support and policy interpretation?	
Policy timeliness	X2	(X2:1) Short-term acute response	1. Does the policy cover short-term (<3 years)?	[27]
		(X2:2) Mid-term	2. Does the policy cover medium-term (3–5 years)?	
		(X2:3) Long-term	3. Does the policy cover long-term development planning (≥5 years)?	
Policy field	X3	(x3:1) Meteorological monitoring and early warning	1. Does the policy cover meteorological disaster monitoring, early warning, and risk assessment?	Text mining
		(x3:2) Medical and health rescue	2. Does the policy cover medical treatment, public health emergency response, health security, and post-disaster disease prevention?	
		(x3:3) Emergency resources and infrastructure	3. Does the policy cover emergency support for transportation, communication, material reserves, and transportation corridors?	
		(x3:4) Cross border collaboration, organization and command	4. Does the policy cover coordination mechanisms, joint command, and inter-departmental collaboration among Guangdong, Hong Kong, and Macao?	
		(x3:5) Science and technology support and capacity building	5. Does the policy cover technological innovation, information platforms, training exercises, and capacity building?	
Policy type	X4	(X4:1) Construction Type	1. Does the policy belong to the long-term investment content of infrastructure construction, capacity cultivation, system construction and so on?	[28]
		(X4:2) Guiding Type	2. Does the policy involve non-mandatory content such as directional guidance, standard advocacy, and opinion reference?	
		(X4:3) Command Type	3. Does the policy involve rigid enforcement such as mandatory constraints, directives, and accountability?	
		(X4:4) Incentive Type	4. Does the policy involve positive-driven content such as resource support, policy incentives, and awards/rewards?	
Policy focus	X5	(x5:1) cross border meteorological disaster monitoring and early warning	1. Does the policy focus on monitoring and early warning of cross-border meteorological disasters?	Text mining
		(x5:2) medical rescue and health emergency	2. Does the policy focus on medical rescue and public health emergency response?	
		(x5:3) collaborative linkage mechanism between Guangdong, Hong Kong and Macao	3. Does the policy focus on the Guangdong–Hong Kong–Macao Greater Bay Area collaborative mechanism?	
		(x5:4) emergency resources and logistics support	4. Does the policy focus on emergency resources and logistics support?	
		(x5:5) emergency capacity building and scientific and technological support	5. Does the policy focus on emergency response capacity building and technological support?	
		(x5:6) organize command and department collaboration	6. Does the policy focus on organization, command, and inter-departmental collaboration?	
		(x5:7) emergency plan and regulations	7. Does the policy focus on emergency plans and regulations?	
		(x5:8) information sharing and communication guarantee	8. Does the policy focus on information sharing and communication support?	
		(x5:9) mobilization of social forces and public participation	9. Does the policy focus on mobilizing social forces and public participation?	
		(x5:10) disaster assessment and disaster reduction and Prevention	10. Does the policy focus on disaster assessment and disaster prevention/mitigation?	
Policy receptor	X6	(x6:1) Guangdong Province, Hong Kong Special Administrative Region and Macao Special Administrative Region	1. Does the policy recipient include the governments of Guangdong Province, Hong Kong Special Administrative Region and Macao Special Administrative Region?	[29]
		(x6:2) Cross regional coordination organization	2. Does the policy recipient include specialized coordinating bodies such as the Leading Group for the Development of the Guangdong–Hong Kong–Macao Greater Bay Area?	
		(x6:3) Professional rescue and medical unit	3. Does the policy recipient include professional medical and health institutions (hospitals, emergency response teams)?	
		(x6:4) Grassroots and social forces	4. Does the policy recipient include grassroots implementing units, social forces, and enterprises?	
		(x6:5) Disaster victims and the public	5. Does the policy recipient include disaster-affected populations and the general public?	
Policy content	X7	(x7:1) Foundation framework completeness	1. Does the policy cover the basic elements of emergency management: disaster type, implementing body, management process, and resource support?	Clustering Network
		(x7:2) Professionalism of medical rescue	2. Does the policy focus on the core of medical and health care, including professional content such as rescue teams, command systems, public health emergency response, and health protection?	
		(x7:3) Response initiation and regional coordination	3. Does the policy clearly define the response triggering conditions and activation time limits, and stipulate the regional linkage and responsibilities boundaries among Guangdong, Hong Kong, and Macao?	
		(x7:4) Institutional mechanism and leadership	4. Does the policy feature institutional innovations in cross-border coordination (such as establishing a joint command mechanism), and clearly define the leadership body and decision-making process?	
		(x7:5) Capacity building and development orientation	5. Does the policy focus on long-term capacity building, including team training, technological support, exercise evaluation, and sustainable development planning?	
Policy guarantee	X8	(x8:1) Financial guarantee	1. Does the policy involve financial security?	[30,31]
		(x8:2) System guarantee	2. Does the policy involve system guarantees?	
		(x8:3) Facility guarantee	3. Does the policy involve infrastructure guarantees?	
		(x8:4) Talent guarantee	4. Does the policy involve talent guarantees?	
		(x8:5) Medical guarantee	5. Does the policy involve medical guarantees?	
		(x8:6) Supervision and management	6. Does the policy involve supervision and management?	
Policy evaluation	X9	(x9:1) Sufficient basis	1. Is the policy basis sufficient?	[25]
		(x9:2) Specific objectives	2. Is the policy objective specific?	
		(x9:3) Detailed planning	3. Is the policy plan detailed?	
		(x9:4) Clarify rights and responsibilities	4. Are the policy’s powers and responsibilities clearly defined?	
Policy disclosure	X10	Policy disclosure	1. Is the policy open to the public?	

**Table 5:** Scores of 26 policies.

Primary Indicators	P1	P2	...	P13	P14	...	P17	P18	...	P25	P26	Average
X1 (Nature of policy)	0.80	0.80	...	1.00	1.00	...	0.80	1.00	...	0.60	0.50	0.74
X2 (Policy timeliness)	0.33	0.33	...	0.67	0.67	...	0.33	0.33	...	0.33	0.67	0.40
X3 (Policy field)	0.80	0.60	...	0.80	1.00	...	0.80	0.80	...	0.80	0.60	0.68
X4 (Policy type)	0.75	0.75	...	0.75	0.75	...	0.50	0.50	...	0.75	0.63	0.55
X5 (Policy focus)	0.60	0.40	...	0.90	1.00	...	0.80	0.90	...	0.45	0.80	0.71
X6 (Policy receptor)	0.80	0.60	...	0.60	1.00	...	0.80	0.80	...	0.60	0.20	0.70
X7 (Policy content)	0.80	0.60	...	0.60	1.00	...	0.80	0.80	...	0.60	0.70	0.65
X8 (Policy guarantee)	0.83	0.83	...	0.83	1.00	...	0.67	0.83	...	0.75	0.33	0.71
X9 (Policy evaluation)	1.00	1.00	...	0.75	1.00	...	0.75	1.00	...	0.88	0.88	0.83
X10 (Policy disclosure)	1.00	1.00	...	1.00	1.00	...	1.00	1.00	...	1.00	1.00	1.00
PMC Index	7.72	6.92	...	7.90	9.42	...	7.25	7.97	...	6.76	6.30	6.95
Policy Text Quality Level	Excellent	Good	...	Excellent	Perfect	...	Excellent	Excellent	...	Good	Good	Good

Note: The “Average” column shows the arithmetic mean of each primary indicator across all 26 policies. The bottom-right “Average” (6.95) is the mean PMC index of the 26 policies. Policy full names are listed in Table 2. Abbreviations: “...” indicates intermediate policies not shown due to space limitations; the complete matrix is available in Supplementary Table S3.

## Data Availability

The data that support the findings of this study are available from the corresponding author, upon reasonable request.

## Supplementary Materials

The following supporting information can be downloaded at: https://www.mdpi.com/article/10.3390/healthcare14121617/s1, Table S1. Summary of samples for evaluating the text quality of policies. Table S2. Top 100 high-frequency word list. Table S3 Scores of 26 policies.
